# ‘Conflict versus Congruence’: A Qualitative Study Exploring the Experience of Gender Dysphoria for Adults with Autism Spectrum Disorder

**DOI:** 10.1007/s10803-019-04296-3

**Published:** 2020-03-13

**Authors:** Rachel S. Coleman-Smith, Richard Smith, Elizabeth Milne, Andrew R. Thompson

**Affiliations:** 1 Department of Psychology, University of Sheffield, Cathedral Court, Sheffield, S1 2LT UK; 2 Sheffield Autism and Neurodevelopmental Service, Michael Carlisle Centre, Sheffield Health and Social Care NHS Foundation Trust, 75 Osborne Road, Sheffield, S11 9BF UK; 3 Present Address: Child Development Psychology Team, Chesterfield Royal Hospital NHS Foundation Trust, Calow, S44 5BL UK

**Keywords:** Autism, Qualitative, Grounded theory, Gender dysphoria

## Abstract

An emergent evidence base indicates a higher prevalence of autism exists amongst people attending gender identity clinics. This qualitative study explored adults’ with autism experiences of coming to understand and address their gender dysphoria (GD). Data were collected and analysed using Grounded Theory. Ten adults with autism and GD undertook semi-structured interviews. A tentative theoretical framework of common processes involved in understanding and addressing GD for individuals with autism was developed. The experience is captured in the core category—*Conflict versus Congruence*. A key finding was the impact of autism as a barrier but sometimes a protective factor in participants’ understanding and addressing GD. Participants appeared to achieve greater personal congruence and wellbeing upon transition. Nevertheless, conflicts remained as they navigated the social world with a continuing fear of hostility and sense of difference due to having two stigmatised identities.

Autism Spectrum Disorder (henceforth referred to as autism) and gender dysphoria (GD) are rare conditions. Population prevalence rates are estimated at 1 in 100 (Brugha et al. 2011) for autism, and 1 in 5000 for GD (Reed et al. 2009). Autism is a neurodevelopmental disorder characterised by impaired social interaction and communication, and restricted and repetitive behaviour, interests or activities (Diagnostic and Statistical Manual of Mental Disorders (DSM-5: American Psychological Association 2013; DSM-5). GD is characterised by distress that accompanies incongruence between experienced or expressed gender and gender assigned at birth (DSM-5). Many people who experience GD want to change their appearance and/or bodily features to be more congruent with their gender identity. Individuals may undertake a ‘transition’ process that can encompass a variety of steps, from social transitioning, to hormone therapy and gender-affirming surgeries.

Despite being rare conditions characterised by distinct diagnostic criteria there is a small but growing body of literature describing their co-occurrence and reporting a significantly higher than expected incidence of autism/autistic traits in gender clinic populations. For instance, 6.4% of children and 9.4% of adolescents referred for GD (De Vries et al. 2010), and 5.5% of adults showed clinically significant levels of autistic traits (Pasterski et al. 2014).

The research base on sexual and/or gender identity in autistic people is limited, comprising principally of case studies; or quantitative research; the voice of the individual with autism is almost absent, instead carer/parent report are relied upon often with a focus on ‘inappropriate behaviour’ and ‘sexual dysfunction’ (Dewinter et al. 2013). A small body of research offer perspectives on the co-occurrence of autism and GD. For instance, indicating GD develops as a “sequel to autism” (Kraemer et al. 2005, p. 295), as an expression of the ‘extreme male brain’ (Di Ceglie et al. 2014); it relates to deficits in Theory of Mind, cognitive rigidity and intolerance of ambiguity (Jacobs et al. 2014); or represents an inherent predisposition toward ‘unusual interests’ (Williams et al. 1996). Many of these studies may be said to be dismissive of individuals’ gender identity experiences by interpreting them as ‘symptoms’ of autism.

Correspondingly, evidence suggests that professionals, services and communities generally fail to “look beyond people’s autism diagnosis” to acknowledge the sexual and gender identity-related needs of individuals with autism (Department of Health [DoH] 2014, p. 22). Individuals with developmental disorders also report difficulty accessing LGBT community spaces due to physical, communication or sensory difficulties and being accepted among non-disabled members (Elderton and Jones 2011). This finding is set within a broader context of governmental reports of adults with autism and their needs continue to be marginalised within services, institutions and the community generally (DoH 2010, 2014).

There is a growing body of research on transgenderism in neurotypical individuals. However, like the autism research, it is predominantly based on researcher led cross sectional investigations despite pleas from transgender individuals to be “qualified not quantified” (Sausa et al. 2007, p. 771). Four Grounded Theory studies of Ekins (1997), Devor (2004) Heistand and Levitt (2005) and Levitt and Ippolito (2014) are exceptions that provide rich accounts of transgender identity development, and identify unique themes relating to this experience, and practical and theoretical implications. However, these studies largely comprise of white, middle-class, non-disabled individuals, and are yet to fully interrogate the significance of other intersecting social identities on individuals’ gender identity. Neurotypical transgender individuals are similarly marginalised and disadvantaged in access to public services and employment; as well as experiencing transphobic hatred and aggression (House of Commons Women and Equalities Committee 2015) They also report marginalisation within LGBT communities (Devor 2015). Those with autism and GD therefore face ‘layered stigma’ (McCann et al. 2016) and ‘double discrimination’ (Elderton and Jones 2011).

Both autism and GD are associated with an increased risk of mental health disorders, most commonly depression and anxiety. An estimated 15% of autistic people (non-transgender) (Balfe and Tantam 2010) and 32% of transgender people experiencing GD attempt suicide (Clements-Nolle et al. 2006). In both populations ‘minority stress’—stigma, prejudice, and discrimination related to holding a social minority identity (Meyer 2003) is a risk factor for mental health problems and suicide (Rosbrook and Whittingham 2010; Winter et al. 2016).

This study sought to build a detailed and contextualised understanding of the experience of gender dysphoria for people with autism. ‘Sensitizing concepts’ gained from familiarity with the literature and personal communications with an expert by experience led the researcher to expect that interpersonal relationships and the social environment may impact on the individual’s experience of GD and gender identity development.

The following research questions were investigated: How does autism impact on the ways in which people understand and address GD? How is this shaped by the social environment?”

## Methods

A qualitative research design was utilised due to its exploratory nature given the dearth of theoretical understanding and research in this area. A Grounded Theory (GT) methodology was chosen as it positions individuals’ actions, experiences and the meaning they attribute to them at its core, whilst seeking to develop a theoretical understanding of these experiences (Charmaz 2014). Thus the approach offered the potential of generating a theoretical framework for the relationship between GD and autism, and the influence of interpersonal experiences and the social environment that is empirically grounded in individual experience.

The original conceptualisation of GT by Glaser and Strauss (1967) is underscored by a realist epistemology—a ‘true’ state of the world exists that may be known, characterised and measured. Glaser and Strauss (1967) postulate that theory is *discovered* by the researcher maintaining objectivity through avoiding importing prior knowledge or assumptions into data generation and analysis. By contrast, the social constructivist version of GT popularised by Charmaz (2014) asserts social reality is “multiple, processual and constructed” (p. 13), and acknowledges researchers’ influence on data collection and interpretation which enables construction of theory formed on shared meaning. The social constructivist GT approach well matched the research question given the approach’s suitability to investigate research questions focusing on psychosocial processes, and its emphasis on individuals’ constructing meaning in relation to their social context (Tweed and Charmaz 2012).

### Participants and Procedure

Opportunity sampling was used to recruit ten participants from a regional gender identity service (*n* = 4); an Autism and Neurodevelopmental service (*n *= 3); an advertisement on a Facebook page for a local support group for transgender people (*n *= 1) and via the expert by experience (*n* = 2).

Participants met the following inclusion criteria: having formal autism and GD diagnoses according to DSM-5/International Classification of Diseases, tenth edition (ICD-10) criteria and sought gender service interventions; aged 18 or over. Individuals at any stage of assessment, intervention or post-treatment were included. Participants were excluded if they: were deemed by clinicians to be ‘high risk’ in terms of suicidal ideation/intent; were non-fluent in English; did not have both GD and autism formal diagnoses.

Although the participants identified as transgender, most endorsed other preferred gender designations (see Table 1). Eight of the participants received their autism diagnosis prior to GD diagnosis. All had socially transitioned, and nine had undertaken some level of physical intervention (hormones and/or surgery). Three participants had completed surgery they desired.

**Table 1:** Demographic and clinical characteristics of the sample

Name (pseudonym)	Age	Preferred gender designation	Sexual orientation	Education level	Age received autism diagnosis	Age received GD diagnosis
Alana	34	Woman	Straight	GCSE	14	32
Alex	18	Man	Gay	GCSE	16	17
Felix	39	Genderqueer	Grey asexual pan-romantic	Masters	34	37
Kate	33	Non-binary transfemine androgynous	Queer	Degree	32	34
Max	28	Man/agender	Bi-sexual	A-level	26	25
Paula	50	Woman	Asexual	NVQ	49	45
Rhianna	31	Woman	Lesbian/asexual	NVQ	14	29
Sam	25	Man	Asexual	NVQ	7	22
Walter	65	Man	Straight	PhD	35	61
Zain	51	Man	Straight	NVQ	40	48

Sexual/gender identity terms used were given by participants

*GCSE* The General Certificate of Secondary Education, an academic qualification, generally taken in a number of subjects by pupils in secondary education in England, Wales and Northern Ireland, *A*-*Level* a qualification in a specific subject typically taken by school students aged 16–18, at a level above GCSE, *NVQ* (National Vocational Qualification) is a work-based way of learning—which is carried out at a college, school, or workplace

### Data Collection

All participants completed a semi-structured interview with the first author. Four participants also consented to second interviews to assist with theoretical sampling and data saturation. In GT the interview schedule evolves over time to explore emerging themes from concurrent data analysis. The initial interview schedule is available in the supplementary materials. Interviews were 70–160 min duration and audio-recorded.

### Analysis

Overall the analysis approach outlined by Charmaz (2006, 2014) was used and is described briefly below. Each interview was transcribed and imported into NVivo 11 qualitative data analysis software (QSR International 2015). Interview data was analysed following each interview allowing the researcher to become sensitised to issues and concepts arising to pursue in subsequent interviews. Analysis began with line-by-line coding of the transcripts, leading to focused coding in later interviews—selecting the most frequently occurring and/or significant earlier codes to sort and synthesise larger segments of data and develop higher-order codes thereby beginning the process of theoretical integration. Data, codes and categories were constantly compared throughout the analytic process. Memo-writing supported the analytic process. Theoretical sampling was undertaken seeking pertinent data to elaborate and hone emerging categories from concurrent data analysis. Credibility checking occurred towards the end of the analysis by asking interviewees whether the emerging categories and the conceptual model represented their experience.

**Fig. 1:**
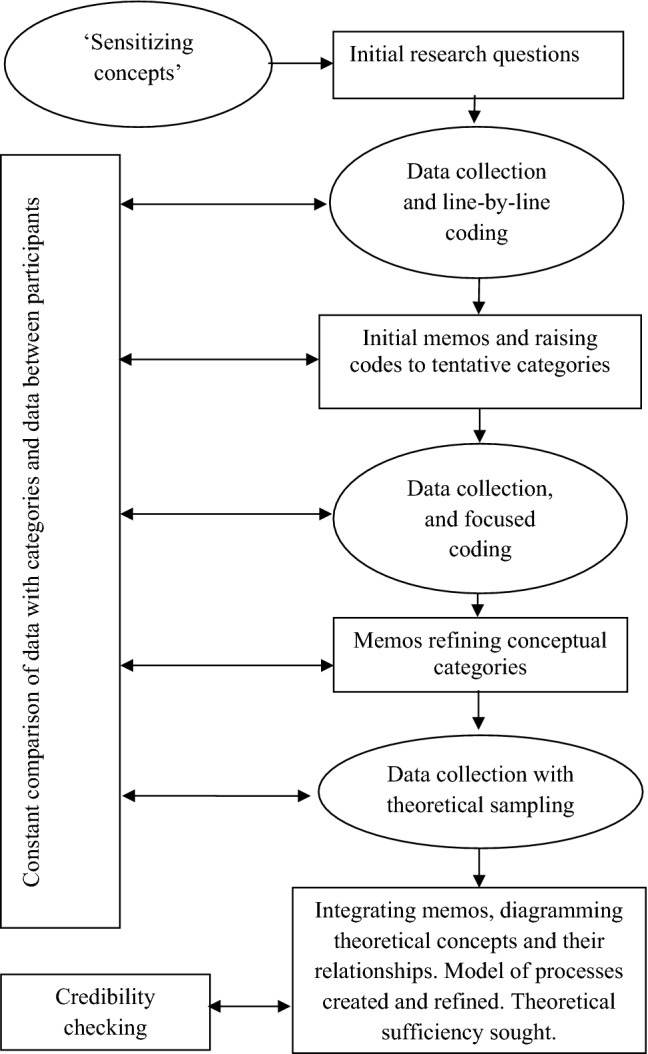
The GT process adapted from Charmaz (2006, p. 11)

In GT researchers generally aim to reach ‘theoretical saturation’—the point at which no new properties of the core categories can be identified. However, some have questioned whether this is possible due to potentially limitless new data, and Dey (1999) suggests the term ‘theoretical sufficiency’ where the researcher claims categories are *suggested* by the data, and this was the approach used here (Fig. 1).

### Ethical and Quality Considerations

Ethical approval was obtained from the University Ethics Committee and the NHS Health Research Authority. Informed consent was gained from all participants included in the study.

Elliott et al.’s (1999) quality guidelines were used to ensure the study’s quality. Their criteria of ‘owning one’s perspective’ and ‘providing credibility checks’, in particular, were given careful consideration. The former involved the first author taking a reflexive approach—identifying her theoretical positioning and personal anticipations, and acknowledging their potential impact on the data collection and interpretation. To aid this process a reflective journal was maintained and discussed with the other authors at key points in the study. ‘Providing credibility checks’ (Elliott et al. 1999) involved discussing emerging categories and conceptual frameworks with participants, and having an independent researcher audit the analysis process.

## Results

Participants’ experiences are described below in terms of categories incorporating illustrative quotations. Participants’ names are pseudonyms.

The analysis built up a framework of common processes involved in coming to understand and address GD, embarking upon social and physical transition, and the impact of autism and the social environment on participants’ experiences. The core category ‘*Conflict versus Congruence*’ captured the key dynamic at play for participants in this process. The categories subsumed within this core category included: ‘*autism as a barrier or protector*’; ‘*restrictive and facilitative social  environments and relationships*’; ‘*feeling* ‘*different*’’; ‘*concealing, and suppressing gender feelings*'; ‘*isolation and poor mental health*’; ‘*percolation of gender feelings*’; ‘*reaching a precipice*’; ‘*social transition*’; ‘*provision of clinical care*’; and ‘*physical transition*’.

As the data was derived (largely) from gender clinic patients who seldom have contact with clinics beyond surgery, most participants had embarked upon physical transition, but only three had completed their desired surgeries. Therefore the predominant focus of the ‘transition’ section is social transition.

As the categories ‘*conflict versus congruence*’; ‘*restrictive and facilitative social environments and  relationships*’ and ‘*autism as a barrier or protector*’ are integral to the common processes (outlined in the remaining categories), they are considered within the detailed exploration of the processes. However, a brief summary of their influence is provided below to orientate the reader.

### Core Category: Conflict Versus Congruence

The core category emerging from the analysis, evident in all participants’ narratives, was ‘*conflict versus congruence*’. This lies at the heart of the experience of GD in adults with autism who have sought to undertake social and physical transition. It connects all categories, suggesting the experience of GD, when one has autism, is one of multi-faceted conflict extending beyond the diagnostic descriptor, comprising: conflict with the body; interpersonal conflict; and psychological or intrapersonal conflict. Resolution of conflict and the pursuit of congruence inform experiences of: ‘*feeling ‘different*’’; ‘*concealing, and suppressing gender feelings*’ and the ‘*percolation of gender feelings*’—the gradual development of understanding of gender identity. Continued suppression of their feelings led some to an experience of ‘*reaching a precipice*’ where only transition may counter the feeling that life is no longer worth living inauthentically. The consequent ‘*social transition*’, is reinforced by ‘*physical transition*’ highlighting the importance of access to appropriate services and the ‘*provision of clinical care*’. Generally, individuals move from high body, inter- and intrapersonal conflict and low congruence earlier in life, to lower conflict and higher congruence as understanding develops and transition is undertaken. However, conflicts occur in social transition, into physical transition and beyond, principally in navigating the social world in a new gender role.

### Autism as a Barrier or Protector

A key theme emerging from this research is that autism was fundamental to the sense of conflict in compounding the challenges associated with GD. Kate: “Autism complicates things…it’s always more complicated with autism”.

Autism impacts significantly upon the process of participants coming to understand and express their authentic gender identities and exacerbates the impact of the social environment. Having autism, a condition affecting social communication, is a *‘barrier’* to interpersonal exploration and sense-making of gender identity. So too, participants described experiencing marginalisation and invalidation of their gender experiences as a result of their having autism. Autism also complicated access to and provision of clinical care due to it impacting on participants’ capacity and confidence to access health services, and the varying accommodation of their needs by services. However, for some few participants *‘autism as a protector’* counteracts fears of negative perceptions, liberating the individual to live authentically according to their gender identity.

### Restrictive and Facilitative Social Environments and Relationships

The impact of the social environment is predominantly restrictive—it hinders understanding, exploring and expressing gender identity, amplifying or maintaining feelings of conflict underpinned by a lack of acceptance of neurodiversity and the dominance of cis-normativity in Western society. Social communication issues complicated accessing interpersonal support. However, accessing supportive relationships/environments was instrumental to enabling understanding and exploration of gender and providing a wider sense of belonging and so increasing personal congruence. These “precious” and “rare” relationships create the necessary preconditions for undertaking transition. Facilitative relationships were commonly with LGBT community members.

### Feeling ‘Different’

Participants described feeling ‘different’ from their earliest memories of childhood. Although initially lacking the concepts or language to understand and articulate their feelings, this related to the interplay of their experience of gender and difficulties related to autism.

Some participants were clear that they experienced being a “different” or “opposite” gender to that assigned to them; whereas others had a more nebulous sense of there being a mismatch between their experienced and assigned gender. Family, peers and teachers used pronouns which did not match participants’ experienced gender, and expected them to behave in ways considered ‘typical’ of their birth-assigned gender. Participants, given their perspective they were not of the gender others saw them as, felt confusion and unease, making their social environment integral to their sense of conflict:

Sam: “I never felt I was a girl and I never…wanted to wear girly clothes or things like that, I always saw myself as being a man…[I thought] everyone else was strange cos they saw me as being a girl.”

As participants aged they became increasingly aware of their difference from other girls and boys—their experience of gender seemingly wasn’t shared with others, which increased their anxiety and sense of ‘difference’. Trans male^1^ participants’ families permitted some flexibility in childhood to behave *more* congruently with their sense of masculinity, although these expressions remained within the socially accepted ‘female’ role of the ‘tomboy’. Trans females^2^ were denied flexibility in experimenting with or expressing femininity. Some participants revealed thoughts of being female to their family which was either ridiculed or chastised.

Zain: “I was always called a tomboy and that was acceptable…because that was someone who was just a sporty female… but if I had known back then about gender dysphoria and raised it, it would have been an absolute no–no”.

School was a major site of conflict—rigidly enforcing traditional gender roles through uniforms, subject selection and the lack of privacy in changing facilities causing anxiety, frustration and magnifying feelings of difference.

Paula: “I wanted…to do girl things like cookery, but…they tried to stop me. Well, I insisted, and they tried to stop me but they didn’t”.

Alex: “Teachers separated us when we were getting changed and I was trying to stay with the boys because I felt awkward with the girls. I didn’t know where to look because - all the bras and things…I’d…feel weird”.

Autism compounded participants’ early feelings of difference and conflict. Some participants felt they had been discriminated against due to having autism, leading them to feel like “second-class citizens”. One participant explained that having autism (and GD) means one sees the world very differently from neurotypical people, but being in the minority your experiences are often invalidated. Gender experiences outside of the norm are similarly nullified.

Zain: “Your experience of the world is really different so it’s always in conflict, so in communication my view and well…I was seen as being in the wrong, but it was like “I can’t be in the wrong”, it was really debilitating!…Add to that seeing gender differently and WHOAH!… Autistic people can end up with mental illnesses because they’re being told their truth doesn’t exist and that’s really scary”.

Social communication difficulties, combined with their different experience of gender were a “double whammy” making social interactions fraught with unease and rendering friendships problematic. Participants reported being “loners” and targets for bullying.

Sam: “The boys didn’t want to play with me and a lot of the girls…And being autistic as well, it’s a double-edge sword…trying to fit in…I don’t think either of them helped with friendships”.

Rihanna eloquently summarised the sense of difference, conflicted selfhood and relationship to others resulting from the interaction of GD, autism and a highly restrictive, stigmatising environment:

Rihanna: “I just didn’t feel I fitted in anywhere, not with my sisters, my brother, other people or EVEN with myself, it’s like everyone was a stranger and I was the strangest of the lot…I’d think…am I just not supposed to exist?!”

### Concealing and Suppressing Gender Feelings

Autism-related social communication difficulties (finding the words to express their gender-related feelings, initiating conversations about them, and predicting others’ responses) combined with the restrictions of a social environment lacking any transgender discourse and intolerance for difference (gender and neurodevelopmental) meant participants felt unable to speak to others of their gender feelings and distress.

Kate: “[Autism] makes it really difficult to say how I feel…I know lots of trans people who don’t have a problem voicing their emotions, and how dysphoria affects them”.

Furthermore, participants hoped that concealing and suppressing their gender feelings and presenting in ways socially expected of their birth-assigned gender may enable them to escape some of the associated conflict and distress. The seeming hopelessness of addressing their difficulties due to their lack of awareness of transition and medical interventions made reducing distress important. Even those with supportive families concealed experiences, fearing wider societal disapproval and discrimination.

Felix: “there’s big conflicts between how you feel and what other people are saying and how you deal with that…you hide who you really are…you mentally push it away…because it’s not good for your mental health to keep banging your head against a brick wall.”

Puberty was an important element of this phase. As puberty dawned, pressure from others to conform to typical gender presentations intensified, even for trans males previously afforded some flexibility.

Felix: “My mum had to persuade me I wasn’t allowed to go around topless in the summer anymore and I was really fed up but felt bound to comply, [due] to gender behaviour expectations again”.

Puberty also intensified GD feelings challenging attempts at ignoring and suppressing them. The bodily changes were sometimes unexpected—as some participants had assumed or fantasized that their body would change to align with their identity—and always devastating, as they sharply brought into focus the conflict between gender identity and body. Most participants described hating their body and not recognising it as their own.

Alex: “Things were appearing where they shouldn’t be. I didn’t like it… I just thought for some reason it wasn’t going to happen to me”.

Rihanna: “More than once I attacked it…one time when I was particularly feeling down I scratched…and scratched. I skinned my arm, mum caught me and sent me to doctors”.

Autism compounded difficulties as participants recognised change and so puberty, was very challenging for them, particularly in the context of their GD. Autism also impeded enactment of expected gender roles through preventing participants from absorbing the requisite cultural norms and schemas:

Alana: “We’re not born with this ‘Mundy^3^’ encyclopaedia or this psychic network that Mundies seem to know naturally what to do, we don’t have that”.

Felix described feeling in a “double bind of trying to ‘pass’ as neurotypical…and also trying to act or ‘pass’ as a woman…I felt like I was in drag.”

### Isolation and Poor Mental Health

Most participants described their anger at others regarding their invalidation, mistreatment and discrimination, but also internalised feelings of hatred and self-blame for their difficulty conforming to cis-normative neurotypical standards:

Rihanna: *“*There’s a lot of things I hate but…nothing can even compare to how much I hate myself, that is the bottom line”.

Distrust of others coupled with low self-esteem and difficulties “passing as normal” led participants to become increasingly withdrawn, further impacting their mental health. Some had suicidal thoughts. Coping mechanisms typically included devoting themselves to schoolwork or other interests, but some participants used alcohol, drugs and/or self-harmed.

### Percolation of Gender Feelings

Despite attempted suppression, participants’ sense of an alternatively-gendered self strengthened over time. Whilst initially outside of conscious awareness, they slowly began a more conscious exploration of their assigned and experienced gender bringing self-understanding and a language for their experiences, and eventually forcing action. Most participants felt their autism diagnosis (for most happening prior to their GD diagnosis) was integral to their developing understanding of their gender identity. This diagnosis was generally a relief, confirming one element of their sense of difference, so enabling them to focus on another. Diagnosis also gave access to support and helped build coping resources for the later challenges of social and physical transition.

Felix: “Now I knew what autism was…Although, what that OTHER thing was, was still a bit unclear.”

Some participants explored their mandated gender status to better understand it and to find a comfortable way of expressing it.

Felix: “I did go through a stage of exploring my femininity…and choosing to go out in the evenings in a skirt and seeing how that felt and…it felt a bit like doing drag… I think exploring femininity gave me a better understanding how I felt my gender to be”.

As Felix indicates, these experiences had the unintended consequence of confirming their lack of fit to their assigned gender. Similarly, two transmen, in the absence of knowledge of transgenderism, felt their experiences related to their sexuality and adopted “butch lesbian” identities which allowed for a temporarily sufficient, expression of their masculinity.

Some participants described insights gained through others witnessing their experienced gender identity, reinforcing its authenticity. As Zain explained, his lesbian partner witnessed the male identity he had not spoken of:

Zain: “[NAME-partner] used to call me her little Italian boy and I really liked that…so yes I suppose…quite similar to my autism diagnosis, these little pieces that just…come into, place…which make sense of so much else of the rest of my life”.

Although participants’ routes to greater understanding of their GD were idiosyncratic, all were in accord that learning about transgenderism was a key moment in their lives. Participants portrayed mixed emotions: relief in having a term to conceptualise their experiences and that they are not alone in feeling as they do, and hope that their distress could be addressed through transition and medical interventions. Many also felt sadness as they realised transgenderism was a stigmatised identity and living an authentic life would be challenging.

Alex: “I saw this Jeremy Kyle episode…[a transgender person] was talking about his childhood…and I thought that sounds a bit familiar…. He just looked like every other man. That’s all I wanted to be…I don’t think it’s what anyone wants to do, go through that, but at least I knew what was wrong”.

Two participants felt their autism diagnosis initially complicated their understanding of GD and gender identity. One felt that receiving the autism diagnosis beforehand led them to conflate the two, distracting them from understanding their gender:

Walter: “Autism became a coat hanger…I hung everything on…it wasn’t until a long time later, when I saw about transitioning I realised these things were clearly about my gender like my breasts…I couldn’t abide them, knowing about them, touching them, that wasn’t sensory sensitivity…it was very specifically gender”.

The other participant explained the “gender-loaded stereotypes” surrounding autism, e.g. ‘the extreme male brain’ theory increased her dysphoria (implying she had a male brain). Her family used this diagnosis to discredit her transgender identity. They assumed her experiences related to autism symptomatology which caused her extreme distress and sometimes to doubt the validity of her dysphoric feelings.

The reverse could also be problematic. A trans male participant whose autism diagnosis came after being diagnosed with GD described focusing on transgenderism as the source of experiences of difference, emotional, interpersonal and body conflict. He felt in retrospect this may be due to a ‘singular focus’ cognitive bias in autism. So autism diagnosis was both facilitative and a barrier.

### Reaching a Precipice

Some participants felt unable to “take on transition”, feeling they lacked resilience due to stressors they had already encountered and/or their circumstances weren’t conducive to change (lacking support at work, in the family, home or in society), they continued to try to conceal and suppress their gender identity.

Felix: “[transition is] about having the courage to assert yourself, but you reach the point of not being able to not assert yourself anymore…I was already being bullied at work, I was struggling due to my Asperger’s…and to do anything that would make me more open to bullying…I couldn’t afford that…I wouldn’t have had the confidence to say ‘right guys I’m not a woman.”

However, as participants’ knowledge of GD and treatment options grew so did their sense of conflict with others who restricted their gender expression, and frustration at themselves for continuing to “live a lie”. Participants’ mental, and in some cases, physical health eroded until they were unable to tolerate suppression and concealment. They reached what Alana described as a “precipice…where the pressure becomes too much, you have reached the limit of your mental endurance, and you think, I’ve got to do something.”

Felix: “I reached that point…everything went completely to hell, my mental and physical health fell to pieces… I’d nothing to lose anymore and…it gave me the courage to say to hell with trying to fit, I’m gonna decide who I am”.

Some participants suggested that autism may compromise tolerance for the distress of GD, leading to a greater need to seek gender support services, and also to increased risk of suicidal ideation:

Alana: “I have been thinking about that [suicide]. I can put on a brave face but sometimes it’s just a bit much…it [tolerating GD] takes a lot of emotional strength, but I hate the whole emotional thing, I don’t handle it well. There’s too much emotional pressure and it does actually require a lot of emotional tolerance…mental endurance, and with autism your endurance isn’t great…so we end up getting help [from gender identity services]”.

By contrast, other participants, based on insight into their preference for preparation and gradual change (some identified as relating to their autism) embarked upon a measured process of planning and building the necessary resources (self-confidence, mental and physical health, further knowledge) to manage transition, working towards achieving a sense of “critical mass” as Sam explained.

Sam: “it was about actually getting yourself to a point where you feel confident enough to be able to do that [transition]…knowing the right way to go about it…getting myself healthy…that helps you to feel much more able to take the first step. I was revving myself up…I suppose”.

### Transition

As the focus of the findings is on individuals’ coming to understand and address GD, the account of transition focuses on its initiation. Transition involves social and physical elements. Social transition involves individuals identifying as their preferred gender identity, presenting and living in greater alignment with it (e.g. through dress, personal pronoun use). Physical transition comprises a range of medical interventions to align the body and gender identity (hormone treatments, surgeries). All participants made both transitions, undertaking social transition first.^4^

### Social Transition

This category differs from preceding ones in that the influence of facilitative relationships and environments came to the foreground in participants’ accounts, providing safety and support to explore gender and self-acceptance. However, autism affects both access to, and the nature of, these relationships and support.

Paramount in participants’ accounts was the importance of connecting with other transgender individuals, with whom transition usually began. Most participants sought out transgender and/or LGBT communities as their certainty increased regarding being transgender and wanting to transition. However, as Kate states:

“Autism obviously makes it quite hard to engage in these relationships in the first place”.

LGBT events often took place in loud and busy pubs, cafés or restaurants, unsuitable for someone with sensory sensitivities. Activities focused on face-to-face unstructured dialogue were challenging due to social communication difficulties. Talking in larger groups particularly induced anxiety:

Kate: “everyone is talking over drinks and it’s a positive atmosphere, and… welcoming…but I felt that I couldn’t engage with it…I get really uncomfortable in that sort of situation…with lots of people having a conversation it’s like ‘Arg!”.

However, structured activities such as committee meetings and workshops/presentations were easier, as were activities where socialising involved ‘doing alongside’ others (e.g. sports). Some participants preferred to access communities online, for instance through chatrooms and social media. Despite accessibility issues, interactions with other transgender people and communities were seen as vital enablers of transition. They facilitated the exchange of practical information and, often for the first time, a sense of belonging:

Kate: “I [made]some friends who were very into being openly feminine, and open about dress and stuff, so that helped me to experiment with dress and presentation…so accepting friend groups help a lot…through them I was able be the person I wanted to be”.

Rihanna: “what really makes me afraid is…having discovered what friends are, there’s this fear of losing them…being alone again”.

Self-blame and internalised transphobia was acknowledged and lessened as they learned through these communities that transphobia is an artefact of gender socialisation and a consequence of peoples’ fear of difference. These experiences led many to support and advocate for transgender people by joining their university or local council LGBT committee, or using social media platforms to educate others on transgender issues. Again, autism impacted on confidence, although their passion to help meant they did all they could. Thus, these communities did much more than facilitate social transition as Sam explained:

“You’re meeting new people…for different reasons and that gives you sense of…belonging, and purpose that you might not have had”.

Emboldened, some participants found other non-LGBT communities offering support and safety to express their gender identity, including poetry groups, conservation organisations and autism support groups. All had diverse membership, so participants felt “at home”.

Participants desired support to transition which necessitated finally disclosing to family and loved ones. However, the fear of rejection (even in supportive relationships) and difficulties related to autism made this highly challenging and often delayed it, particularly for those still living with parents due to their additional support and accommodation needs.

Alana: “My personal experience has always been positive…no fights, no rows…I know X [name], I can tell her anything but I wasn’t even sure about that because of the social perception…there was still that fear”.

Responses to disclosure varied, as did their impact on transition. For some with historically discordant family relationships, disclosure resulted in their predicted rejection and, autism was often used to discredit their gender experiences. This did not alter decisions to transition.

Sam: “I’ve always enjoyed collecting toys; [my father] said “if you’re still playing with toys…you’re not mature enough to make these decisions [about gender/transition]”…he thinks because of my interests I couldn’t know about my gender…when your gender is wrong it’s pretty clear!”

Most participants spoke of relief having socially transitioned, describing it as when their “life properly began”. In being more congruent in their gender expression they felt “authentic” which improved their self-confidence and wellbeing. Participants’ body conflict also decreased somewhat, and those who identified with the gender binary felt they fitted in more:

Alex: “I feel like I fit into a group a bit better…I can look in the mirror and I look more like a man, at least with my clothes on”.

Participants’ accounts portrayed a positive cycle developing as Alana explains:

“Since I’ve been like this, I’ve been happier…The more happy I am, the more engaged I am and the more I want to engage…the more I do and…aren’t misread, the happier I am!”.

Participants’ increased intrapersonal and body congruence meant they felt increasingly confident to engage with others and life, especially when others saw them as they saw themselves, which reinforced wellbeing. However, this virtuous cycle was fragile given the threat of being seen as their birth-assigned gender and abused. One participant had experienced physical abuse; most others verbal abuse, especially transphobic comments. The effect was devastating and could lead to a return to concealment, isolation and poor mental health.

Alex: “At first your confidence is through the roof…then it wouldn’t take much for it to just crumble…At first I found it a lot easier to make friends, so I had more friends. Then after a while it went away…a few random comments can really get to me, I get really paranoid about it…I don’t go out as much”.

Most participants felt autism complicated social transition through their not having an intuitive, neurotypical “encyclopaedia” of presentation making it more likely they were misread. Furthermore, some participants felt less able to ‘pass’ as neurotypical as they hadn’t learned how to “perform that role” in relation to a new gender presentation and anxiety about being misread made their autistic behaviours more pronounced:

Felix: “I don’t feel like I’m fully socially transitioned because I am experiencing more autism barriers than I was as a woman…I had thirty odd years to learn how to pretend to be female…and I’m still trying to come up with new strategies to be consistently read as male because expectations are different…Autism makes this slow. I’m also being read more as autistic because I’ve not learned the disguises for someone now read as male…My confidence is affected…so more autistic behaviour, like stim[ing] creeps out.”

Practice in interacting with others was important to reinforce social transition, but heightened anxiety meant it was often a battle not to withdraw again. Contrastingly, a number of participants suggested autism facilitated transition, describing autistic people as falling into two types:

Zain: “…there’s us that are oblivious to what other people think of us…; and there’s those who worry about what everybody’s thinking…I fit into the category of I’m not bothered, which is a blessing, so I’ve not had any negative experiences…or…possibly if there has been something said and done I’ve missed it”.

For some then, their “obliviousness” to others’ perceptions may protect them from the experience of being misread, thus facilitating transition. Furthermore, some felt their unavoidable difference due to autism enabled them to express their authentic gender identity. They had come to accept social rejection and placed little worth on others’ opinions.

Zain: “I’m quite happy with, my shield of ‘this is who I am’, if I don’t actually do the ‘right’ social male thing tough…I’m used to not fitting in anyway”.

## Provision of Clinical Care

Gender services were a crucial facilitative environment/relationship in undertaking transition. However, services and clinicians’ attitudes and variable adaptability to autism could compound autism-related difficulties in participants accessing support. Participants proposed that having autism may increase anxiety about attending gender services beyond what neurotypical people may experience. Some attempted to manage their anxiety by extensively researching the assessment process, but would have preferred more guidance from services. For some these issues delayed their first medical appointment.

Alex: “it’s [autism] just made it more a nerve-wracking experience…what’s going to happen, what’s going to be said, what will happen next?…I nearly didn’t go”.

Unfortunately, two participants felt they were denied treatment from specialist gender services because of their autism.

Rihanna: “They didn’t say directly but I felt because I’d got Asperger’s they wouldn’t really take me seriously…I spiralled down…and couldn’t pick myself up.”

Upon accessing services some participants experienced distress due to a lack of transparency and predictability in the process, and timescales of assessment and treatment. Participants feared being denied treatment due to not being able to give the ‘right’ or appropriate responses, reflecting communication difficulties.

Kate: “I get the impression they’re looking for very specific comments [about my body] but I just don’t know…it’s difficult to explain”.

While desiring body change, the same change provided a trigger for anxiety which was attributed to autism. Clinicians allowing time for discussion of concerns and implementing stepped change was helpful:

Zain: “…he discussed all the reasons I was uncomfortable [with hormone therapy]… the pros and the cons and spent ages, so in the end I was like “oh okay I’ll try the gel”…cos if I got anxious I could wash it off…then I moved onto injections…doing that in small increments I was okay”.

The participant who didn’t receive his autism diagnosis until after his GD diagnosis felt this hindered the assessment and treatment process as the service wasn’t able to adapt to his needs and he wasn’t able to anticipate the additional difficulties he may encounter in transition. Specifically, he had not expected to experience a “second puberty” and how profoundly distressing this would be:

Max: “I did loads of research and prepared…they’d ask questions like “so you know about the changes that will come with hormones” but I hadn’t really processed that meant those things would happen to me…puberty was already a really awful time and I had it again”.

### Physical Transition

Decisions around physical transition were shaped by the need to reduce dysphoria by aligning body and gender identity, but also to reduce the likelihood of being misread and the associated threat of discrimination and harm. Kate’s comment illustrates this and how it came into conflict with her values.

Kate: “I’m hoping transition will alleviate some of the social anxieties around gender roles and expectations…though it leads me back to conflict…transphobia and gender discrimination shouldn’t exist…the idea of passing into an oppressive system is really difficult for me, there shouldn’t be that oppression in the first place!”

Despite such concerns, this was the only option participants felt was open to them which would improve their lives-, a “lesser of two evils”.

Upon embarking upon transition, male-identifying participants reported rapid improvements in dysphoria and social anxiety, as taking hormones and growing facial hair made them feel instantly recognisable as masculine. The trans women felt physical changes were slower to achieve and for some facial masculinisation, in particular, continued to be a source of discomfort with their appearance. Overall, accounts of embarking on physical transition portrayed a reinforcement of social transition as the more congruent participants felt in their body and gender presentation, the more they engaged, providing opportunities for validation of their identity, and so enhancing their wellbeing. Walter nicely summed up the experience of increased personal congruence:

“The more I’ve moved into this role as a man ahh I have never felt so whole!”

All participants reported experiencing personal growth, greater self-awareness, resilience and empathy from overcoming the challenges they experienced.

Sam: “I have mixed feelings about being trans…I’m not glad that I was born a girl, but I’m glad it gives me a great understanding of myself and being able to empathise with people,… trans people…if I was someone looking from the outside I might not be as sympathetic or supportive…I think it makes me a better person for having to work to be the person I am”.

As Sam’s quote indicates, although things were much improved for participants, most were keen to impress all is not resolved.

Felix: “People think you go down a certain path and you’re done [physical transition], you live happily ever after but it’s a life-long process of navigating your environment”.

Despite personal growth, increased body-gender congruence, and interpersonal congruence through ‘fitting into a group’, and finding belonging among trans people and others, participants portrayed an enduring and “ingrained” sense of being ‘different’ or “fake”. The ‘fakeness’ has a dual nature relating to their enactment of a new gender role which is compromised by autism-related difficulties and invalidation by others; and their attempted enactment of neurotypicality. The ‘difference’ is an enduring artefact of the double discrimination endured and internalised. This limited their sense of identity congruence and self-acceptance, and with that came an expectation of rejection and harm from others:

Kate: “I find it hard to think of myself as ‘authentic’ however I present myself. I feel fake. I really hope that will change…but that feeling is quite deeply ingrained. I will probably have to live with it.”

## Discussion

To the best of our knowledge, this study is the first to examine the perspectives of adults with autism regarding their experience of GD, and the analysis provides a tentative model of common processes involved for participants in coming to understand their gender identity and address GD. It illustrates the significance and impact of autism as both a ‘barrier’ and ‘protector’ to these processes as well as the influence of the social environment as ‘restrictor’ or ‘facilitator’. Autism complicated the process of understanding and addressing gender concerns and accessing supportive and facilitative relationships. Once transition had occurred, autism also impacted on participants’ enactment of their authentic gender identities leaving some feeling vulnerable to being mis-gendered and/or harmed. Notwithstanding this, autism also offered some a sense of protection from societal expectations and prejudice either as participants were oblivious to them, or they had habituated to the impact of discrimination related to having autism and so defied mores relating to gender.

The findings resonate with the few qualitative studies examining transgender identity development in the neurotypical population, which also use a GT approach: Devor (2004), Levitt and Ippolito (2014), Ekins (1997); and Heistand and Levitt (2005). Models share initial periods of gender identity confusion and distress; increasing social pressures to conform to cis/heteronormative ideals; a gradual testing out of gender identities, finding an identity that fits; and highlight the centrality of accessing gender-affirming social groups in facilitating gender exploration and self-acceptance. However, previous models differ in that although the influence of discrimination and threat of harm on gender expression is evident, it appears to play a less prominent role than in the current study. Furthermore post-transition identity integration and self-acceptance, appears more fully actualised in previous studies. In Heistand and Levitt (2005, p. 74) participants’ “negative feelings [about their identity] were dispelled and being butch became a prideful identity”; in Devor (2004) participants achieve “serene self-acceptance” and “gender euphoria” (p. 63); in Levitt and Ippolito (2014) participants achieve “authenticity” and in Ekins’ (1997) identity is “consolidated”, although both studies acknowledge presentation involves compromise due to need to manage risk of harm. Whilst the current study’s participants achieved increased congruence and wellbeing, there was an enduring internal conflict as outlined above attributed to having autism in combination with GD. Therefore, study comparison indicates a common identity development process, complicated by autism. Social communication impairments thwart understanding, exploring and presenting gender identity; and the ‘layering of stigma’ (McCann et al. 2016) appears to increase fear of discrimination, ultimately complicating the forming of positive gender identity and self-acceptance. Time elapsed since transition may play a role in identity integration, though previous studies did not report this data.

The findings mirror qualitative research exploring sexual identity development in people with intellectual disabilities (ID). Here again, individuals face the “double disadvantage” of heteronormativity and ableism in forming a positive sexual identity (Wilkinson et al. 2015, p. 102). ID overshadows their sexual identity which is ignored, invalidated or pathologised by family, services and society generally, limiting opportunities for identity exploration and leading to concealment and shame (Burns and Davies 2011).

A key finding was the importance of affirming relationships and social support to transition and well-being, something supported by the wider literature on neurotypical transgender individuals. Positive identification with one’s social group [collective self-esteem (Crocker and Luhtanen 1990)] has been found to be negatively correlated with psychological distress in transgender people (Sánchez and Vilain 2009). Similarly, family support is associated with higher self-esteem and life satisfaction in transgender people (Erich et al. 2008) and social support, generally, has been found to predict psychological functioning following gender-confirming surgery (Davey et al. 2014). Social support has also been found to be significantly positively related to quality of life in autistic people (Renty and Roeyers 2006) and can protect against the emotional impact of victimisation (Humphrey and Symes 2010). However, individuals’ with autism difficulty accessing social support due to ostracism, social communication impairments and the inaccessibility of social environments evidenced in the current study finds ample support in the existing literature (e.g. Müller et al. 2008; Smith and Sharp 2013).

The findings highlighted that autistic people, fearing social responses and struggling to understand and express emotions, will conceal their gender concerns often until life becomes unbearable. The toll of concealment, and, therefore, lack of support for GD, is tremendous. Professionals working with individuals with autism need to proactively address sexuality and gender issues to ensure individuals access the support they need.

Services may support access to supportive communities through setting-up transgender peer mentoring programmes, or support groups which are inclusive of those with autism. The current study’s participants specified conducive group formats, including structured activities based on a topic rather than unstructured face-to-face dialogue. Online peer support groups may also be a useful avenue for supporting this population given the current study’s finding supported by wider growing evidence of its benefits to wellbeing (Griffith et al. 2012).

Experiences of gender clinics indicated improvements could be made to enhance accessibility. Some individuals perceived staff prejudice relating to autism, and experienced anxiety due to inadequate information on the assessment and treatment process. This echoes the wider literature on healthcare experiences of autistic people (e.g. DoH 2010; Nicolaidis et al. 2015). Asking patients about their needs would be a first step in ensuring they are better accommodated. The value of providing additional time to explain assessment and treatment processes and to provide reassurance was indicated in the findings. Given the high prevalence of autism in gender clinic patients, support for clinicians to meet this population’s needs is crucial. Involving experts by experience would enhance the training, with the autism community having made clear they want to be involved in service development (Griffith et al. 2012).

There are a number of limitations to the present study. Participants were able to provide verbal accounts of their experiences in interviews, and would fit the profile of the prior diagnosis of Asperger’s syndrome or ‘high-functioning’ autism. This potentially limits the findings’ representativeness and reduces the generalisability of our findings to individuals with greater functional challenges, including social anxiety. Future studies may consider other ways of involving people representative of the wider population of autistic people which suit their abilities and communication preferences (e.g. internet-based interviews or using assisted communication).

Furthermore, the small sample consisting of Caucasian individuals who had accessed NHS services in the UK, limits transferability to individuals residing in countries with different notions of gender, or where they must pay for gender-confirming treatment. All participants in the study were adults, and therefore our findings do not speak to the experiences of autistic children and adolescents with GD. In addition, the study charted individuals’ experiences up to embarking upon physical transition, dictated by gender services supporting people mainly until surgery and the challenges of accessing participants who have moved beyond physical transition in this very niche population. Future studies may consider experiences beyond this timeframe and the balance of ongoing conflict and congruence as time passes beyond completion of physical transition.

Data saturation was not achieved and the physical transition category, in particular, was comparably less thick and rich (Fusch and Ness 2015) due to so few of the participants having completed this process. This potentially limits confidence in the robustness of the final conceptual model.

## Conclusion

The findings highlight this population’s vulnerability to stigma, discrimination, isolation, low self-worth and poor mental health as a ‘minority within a minority’. Transition brings relief from the toll of suppression, increased well-being, greater engagement with others and improved quality of life. However, in the restrictive social environment, living a more authentic life involves compromises for many. Conflicts remain as participants navigate the social world with an enduring fear of hostility and sense of difference and ‘fakeness’ due to having two stigmatised identities. Services should be proactive in discussing gender issues with autistic people to ensure their needs are addressed sooner. It is crucial that social support is facilitated for this population, particularly connections with other transgender people.

## Appendix

### Interview Schedule

- How old are you?
- What is your preferred way to identify your gender?
- How would you describe your ethnicity?
- What is your highest level of education achieved?
- Are you employed?
- What is your relationship status?
- What term would you use to describe your sexuality?
- At what age were you when you were diagnosed as being on the autistic spectrum?
- Do you have any medical diagnosis, besides autism and gender dysphoria?
- Are you currently taking any regular medication (aside from those connected to gender transitioning)?
- When did you first attend gender identity services?

### Initial Open-Ended Questions about GD

- How do you experience your gender?
- What does ‘gender dysphoria’ mean to you?

#### Further Questions

- Tell me about when you first got the sense that your gender identity did not match your gender assigned at birth.
- What happened to these feelings over time?
- Are there any moments or experiences in your life which stand out as important in your experience of GD?

### Relationship Between GD and Autism

- How do autism and GD interact together (if they do at all)?
- What impact has having autism had on your experience of GD?
- What impact has autism had on your addressing your GD?

#### Follow Up

Are there ways in which having autism has helped/protected you in your experience of GD?/Been a barrier?

### Interpersonal Relationships and the Social Environment

- What impact have your relationships/experiences with others had on your understanding of GD and your gender identity development?
- How has your autism and having gender dysphoria influenced your relationships with: (a) family/loved ones; (b) services; (c) LGBT community; (d) society in general (neurotypical, cisgender people).

#### Further Questions

Tell me about how other people have responded to your gender dysphoria.

How have you coped with challenges from others/the environment?

### Relationship with Body

- How would you describe your relationship with your body?
- Has this changed over time?

### Ending Questions

- Is there anything else that you think I should know that would help me to understand living with GD as someone with autism?
- How did you find being interviewed? What if anything would have improved your experience?

## Footnotes

1 Individuals assigned female at birth whose gender identity is male/masculine.

2 Individuals assigned male at birth whose gender identity is female/feminine.

3 Neurotypical people.

4 A requirement for NHS gender-affirming surgeries (NHS England (2013) *Interim Gender Dysphoria Protocol and Service Guideline*).

## References

[CR1] American Psychological Association (2013). Diagnostic and statistical manual of mental health disorders: DSM-5.

[CR2] Balfe M, Tantam D (2010). A descriptive social and health profile of a community sample of adults and adolescents with Asperger syndrome. BMC Research Notes.

[CR4] Brugha, T. S., McManus, S., Bankart, J., Scott, F., Purdon, S., Smith, J., … Meltzer, H. (2011). Epidemiology of autism spectrum disorders in adults in the community in England. *Archives of General Psychiatry*. 10.1001/archgenpsychiatry.2011.38. 10.1001/archgenpsychiatry.2011.38 21536975

[CR5] Burns J, Davies D (2011). Same-sex relationships and women with intellectual disabilities. Journal of Applied Research in Intellectual Disabilities.

[CR100] Charmaz K (2006). Constructing grounded theory.

[CR6] Charmaz K (2014). Constructing grounded theory: A practical guide through qualitative analysis.

[CR7] Clements-Nolle K, Marx R, Katz M (2006). Attempted suicide among transgender persons: The influence of gender-based discrimination and victimization. Journal of Homosexuality.

[CR8] Crocker J, Luhtanen R (1990). Collective self-esteem and in-group bias. Journal of Personality and Social Psychology.

[CR9] Davey A, Bouman WP, Arcelus J, Meyer C (2014). Social support and psychological well-being in gender dysphoria: A comparison of patients with matched controls. The Journal of Sexual Medicine.

[CR102] Dey I (1999). Grounding grounded theory: Guidelines for grounded theory inquiry.

[CR101] Di Ceglie, D., Skagerberg, E., Baron-Cohen, S., & Auyeung, B. (2014). Empathising and systemising in adolescents with gender dysphoria. *Opticon*, *1826*. 10.5334/opt.bo.

[CR10] De Vries AL, Noens IL, Cohen-Kettenis PT, van Berckelaer-Onnes IA, Doreleijers TA (2010). Autism spectrum disorders in gender dysphoric children and adolescents. Journal of Autism and Developmental Disorders.

[CR11] Department of Health. (2010). *Fulfilling and rewarding lives: The strategy for adults with autism living in England.* http://webarchive.nationalarchives.gov.uk/20130107105354/http://www.dh.gov.uk/en/Publicationsandstatistics/Publications/PublicationsPolicyAndGuidance/DH_113369.

[CR12] Department of Health. (2014). *Think autism: Fulfilling and rewarding lives, the strategy for adults with autism in England: An update.* https://www.gov.uk/government/uploads/system/uploads/attachment_data/file/299866/Autism_Strategy.pdf.

[CR13] Devor AH (2004). Witnessing and mirroring: A fourteen stage model of transsexual identity formation. Journal of Gay & Lesbian Psychotherapy.

[CR14] Devor, A. H. (Writer/Interviewee). (2015). Transforming gender. In M. Guerre (Producer). *Doc Zone*. Ottawa, ON: Canadian Broadcasting Corporation.

[CR15] Dewinter J, Vermeiren R, Vanwesenbeeck I, Nieuwenhuizen C (2013). Autism and normative sexual development: A narrative review. Journal of Clinical Nursing.

[CR16] Ekins R (1997). A grounded theory approach to cross-dressing and sex-changing: Male femaling.

[CR17] Elderton A, Jones C (2011). Finding a safe place to explore sexual identity. Learning Disability Practice.

[CR18] Elliott R, Fischer CT, Rennie DL (1999). Evolving guidelines for publication of qualitative research studies in psychology and related fields. British Journal of Clinical Psychology.

[CR19] Erich S, Tittsworth J, Dykes J, Cabuses C (2008). Family relationships and their correlations with transsexual well–being. Journal of GLBT Family Studies.

[CR20] Fusch PI, Ness LR (2015). Are We There Yet? Data saturation in qualitative research. The Qualitative Report.

[CR103] Glaser BG, Strauss AL (1967). The discovery of grounded theory: Strategies for qualitative research.

[CR21] Griffith GM, Totsika V, Nash S, Hastings RP (2012). I just don’t fit anywhere: Support experiences and future support needs of individuals with Asperger syndrome in middle adulthood. Autism.

[CR22] Heistand KR, Levitt HM (2005). Butch identity development: The formation of an authentic gender. Feminism & Psychology.

[CR23] House of Commons Women and Equalities Committee. (2015). *Transgender equality.* https://www.publications.parliament.uk/pa/cm201516/cmselect/cmwomeq/390/390.pdf.

[CR24] Humphrey N, Symes W (2010). Perceptions of social support and experience of bullying among pupils with autistic spectrum disorders in mainstream secondary schools. European Journal of Special Needs Education.

[CR25] Jacobs LA, Rachlin K, Erickson-Schroth L, Janssen A (2014). Gender dysphoria and co-occurring autism spectrum disorders: Review, case examples, and treatment considerations. LGBT Health.

[CR26] Kraemer B, Delsignore A, Gundelfinger R, Schnyder U, Hepp U (2005). Comorbidity of Asperger syndrome and gender identity disorder. European Child and Adolescent Psychiatry.

[CR27] Levitt HM, Ippolito MR (2014). Being transgender navigating minority stressors and developing authentic self-presentation. Psychology of Women Quarterly.

[CR28] McCann E, Lee R, Brown M (2016). The experiences and support needs of people with intellectual disabilities who identify as LGBT: A review of the literature. Research in Developmental Disabilities.

[CR29] Meyer IH (2003). Prejudice, social stress, and mental health in lesbian, gay, and bisexual populations: Conceptual issues and research evidence. Psychological Bulletin.

[CR30] Müller E, Schuler A, Yates GB (2008). Social challenges and supports from the perspective of individuals with Asperger syndrome and other autism spectrum disabilities. Autism.

[CR104] NHS England. (2013). Interim gender dysphoria protocol and service guideline 2013/14. NHS England Medical Directorate. https://www.gires.org.uk/wp-content/uploads/2017/03/int-gend-proto.pdf.

[CR31] Nicolaidis, C., Raymaker, D. M., Ashkenazy, E., McDonald, K. E., Dern, S., Baggs, A. E., … Boisclair, W. C. (2015). “Respect the way I need to communicate with you”: Healthcare experiences of adults on the autism spectrum. *Autism*. 10.1177/1362361315576221. 10.1177/1362361315576221 PMC4841263 25882392

[CR32] NVivo 11 (QSR International, 2015). Computer software. http://www.qsrinternational.com.

[CR33] Pasterski V, Gilligan L, Curtis R (2014). Traits of autism spectrum disorders in adults with gender dysphoria. Archives of Sexual Behaviour.

[CR34] Reed, B., Rhodes, S., Schofield, P., & Wylie, K. (2009). *Gender variance in the UK: Prevalence, incidence, growth and geographic distribution*. http://www.gires.org.uk/employment/information-on-prevalence-and-incidence.

[CR35] Renty JO, Roeyers H (2006). Quality of life in high-functioning adults with autism spectrum disorder: The predictive value of disability and support characteristics. Autism.

[CR36] Rosbrook A, Whittingham K (2010). Autistic traits in the general population: What mediates the link with depressive and anxious symptomatology?. Research in Autism Spectrum Disorders.

[CR37] Sánchez FJ, Vilain E (2009). Collective self-esteem as a coping resource for male-to-female transsexuals. Journal of Counselling Psychology.

[CR38] Sausa LA, Keatley J, Operario D (2007). Perceived risks and benefits of sex work among transgender women of colour in San Francisco. Archives of Sexual Behaviour.

[CR39] Smith RS, Sharp J (2013). Fascination and isolation: A grounded theory exploration of unusual sensory experiences in adults with Asperger syndrome. Journal of Autism and Developmental Disorders.

[CR40] Tweed A, Charmaz K, Harper D, Thompson A (2012). Grounded theory methods for mental health practitioners. Qualitative research methods in mental health and psychotherapy.

[CR41] Wilkinson VJ, Theodore K, Raczka R (2015). ‘As normal as possible’: Sexual identity development in people with intellectual disabilities transitioning to adulthood. Sexuality and Disability.

[CR42] Williams PG, Allard AM, Sears L (1996). Cross-gender preoccupations in two male children with autism. Journal of Autism and Developmental Disorders.

[CR43] Winter S, Diamond M, Green J, Karasic D, Reed T, Whittle S, Wylie K (2016). Transgender people: Health at the margins of society. The Lancet.

